# Supplementary material for: Are Matrix Metalloproteinase-9 and Tissue Inhibitor of Metalloproteinase-1 Useful as Markers in Diagnostic Management of Children with Newly Diagnosed Ulcerative Colitis?
Source: J Clin Med. 2022 May 9;11(9):2655. doi: 10.3390/jcm11092655 (PMC9103541; doi:10.3390/jcm11092655)
Supplement: Supplementary file 1 [file jcm-11-02655-s001.zip › jcm-1624044-supplementary.pdf]

Table **S1**. Immunochistochemical staning of MMP-9 and TIMP-1 in biopsy samples of patients with Colitis Ulcerosa (CU) and control Group (Ctr).

	MMP-9 Staining				TIMP-1 Staining			
	Intensity	Extent	Sum score	Expression in glandular epithelium	Intensity	Extent	Sum score	Expression in glandular epithelium
CU	2 (1-3)	4 (1-4)	6 (2-7)	70% (20-100%)	3 (1-3)	4 (2-4)	7 (3-7)	100% (30-100%)
Ctr	0.5 (0-1)	0.5 (0-1)	1 (0-2)	1.5% (0-10%)	0 (0-1)	0 (0-5)	0 (0-2)	0% (0-5%)

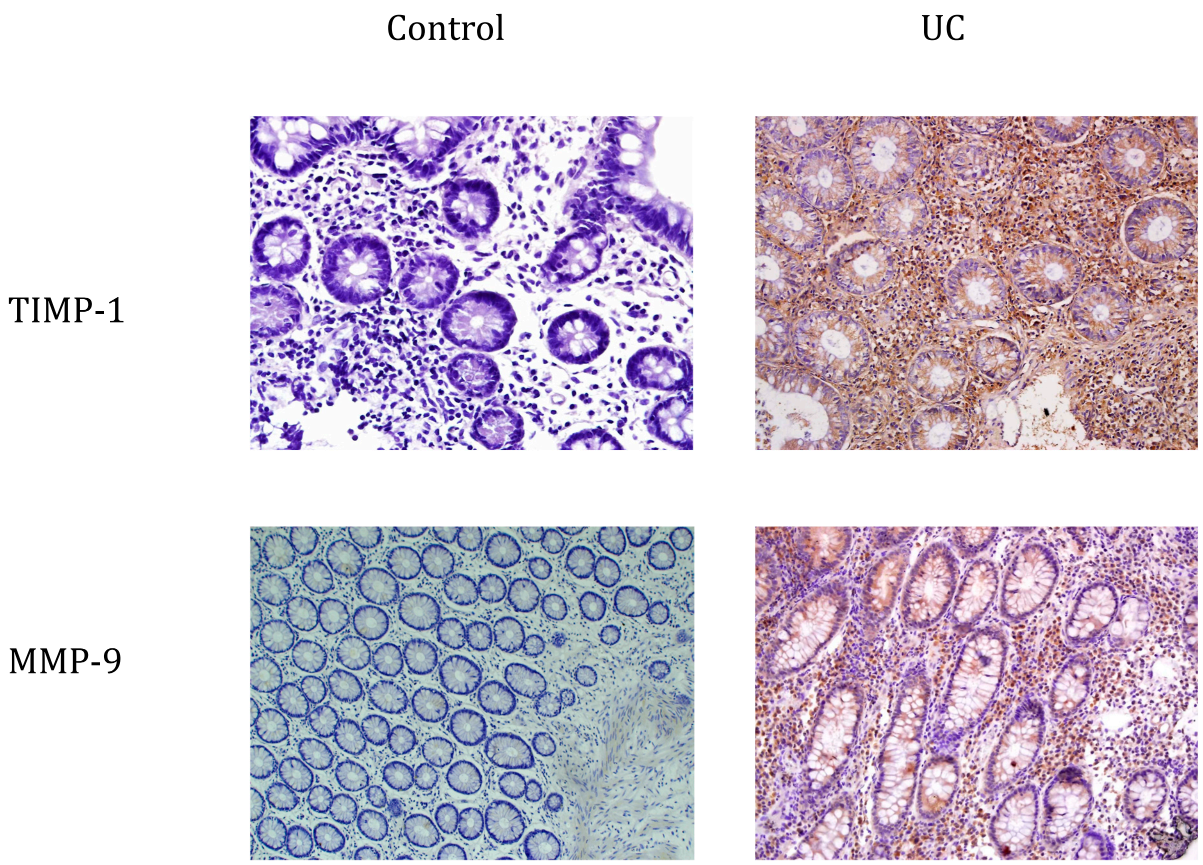

Figure S1: Immunochistochemical staining shwoing mucosal expression of TIMP-1 and MMP-9 in UC and Control group
